# The beneficial impact of pomegranate oil nanoemulsion on the quality of cryopreserved bovine sperm: antioxidant and anti-apoptotic effects

**DOI:** 10.5713/ab.25.0067

**Published:** 2025-04-11

**Authors:** Fatma Mohsen Shalaby, Soha A. Hassan, Ali A. El-Raghi, Fatemah Enad Alajmi, Mohamed G. Alfayoumi, Kandil Abd El Hai Attia

**Affiliations:** 1 Department of Zoology, Faculty of Sciences, Mansoura University, Dakahlia, Egypt; 2 Department of Biology, Faculty of Sciences, King Khalid University, Abha, Saudi Arabia; 3 Department of Biotechnology, Faculty of Applied Health Sciences, October 6 University, Giza, Egypt; 4 Department of Animal, Poultry and Fish Production, Faculty of Agriculture, Damietta University, Damietta, Egypt; 5 Department of Biology, College of Science, University of Hafr Al Batin, Hafr Al Batin, Saudi Arabia; 6 General Department of Forensic Science and Criminology, Dubai Police General, Dubai, UAE; 7 Evaluation of Natural Resources Department, Environmental Studies and Research Institute, University of Sadat City, Minufiya, Egypt

**Keywords:** Antioxidant, Apoptotic Genes, Bovine Semen Cryopreservation, Pomegranate Oil Nanoemulsion, Ultrastructure

## Abstract

**Objective:**

This study was conducted to investigate the effect of pomegranate oil nano-emulsion (PO-NE) supplementation in semen extender on semen quality, redox status and apoptotic genes of cryopreserved bovine semen.

**Methods:**

Seven proven fertility Holstein Friesian bulls (4 to 6 years) were involved, and the semen samples were collected using the artificial vagina method. Semen was pooled and cryopreserved in tris extender containing PO-NE at 0 (PO-NE0), 1 (PO-NE1), 2 (PO-NE 2) and 4 μg/mL (PO-NE4), respectively.

**Results:**

Incorporating 2 or 4 μg/mL PO-NE into freezing media significantly increased sperm progressive motility, viability, membrane integrity, and kinematic parameters. Furthermore, the aforementioned two treated concentrations demonstrated superior anti-oxidative activities (total antioxidant capacity and super oxide dismutase) and higher nitric oxide levels compared to the control group (p<0.05). The levels of hydrogen peroxide, malondialdehyde, and Nuclear Factor-Kappa B were notably lower in the PO-NE4 treated group compared to the control group. The addition of 2 or 4 μg/mL of PO-NE to the freezing media significantly downregulated pro-apoptotic genes (*caspase 3, Bax*), while significantly induced the expression of anti-apoptotic gene Bcl2. The addition of PO-NE preserved plasma membrane and acrosome integrity and maintained the ultrastructure of sperm, contrasting with PO-NE0, which exhibited the most damage.

**Conclusion:**

Supplementing the bovine freezing extender with 2 or 4 μg/mL of PO-NE enhanced post-thawed sperm characteristics by reducing oxidative stress, improving antioxidant indices and apoptotic genes expression, and preserving the ultrastructure integrity of frozen-thawed cattle sperm.

## INTRODUCTION

Cryopreservation of gametes is a vital technique for artificial insemination (AI), enabling the economical transfer and distribution of gametes with desirable genetic traits worldwide. Recently, Holstein Friesian bulls have experienced a progressive increase in AI applications, especially in countries where intensive breeding systems are common [1]. According to previous reports, the use cooled semen for a brief duration (36 hours) enhances prolificacy and fertility rates [2]. On the flip side, AI with cryopreserved sperm has shown reduced fertility and prolificacy rates in Friesian bulls [1]. Cryopreservation is known to impact sperm adversely through various mechanisms, causing sub-lethal damage and alterations that contribute to a shortened lifespan of sperm cells [1]. Throughout cryopreservation, several stress factors affect sperm functionality, including temperature decrease, ice crystal formation, increased osmolality, and the generation of oxidative stress (OS) [3]. The freezing-thawing cycle triggers excessive OS, leading to denaturation of lipid and protein structures, decrease motility and viability, damages acrosomes, mitochondria, and tails, and increases DNA fragmentation in mammalian spermatozoa [4]. Additionally, the freezing and thawing procedures increase intracellular calcium levels and membrane fluidity leading to precipitation and various physiological instabilities within the cell [4,5]. Sperm cells are particularly susceptible to damage caused by OS due to their high polyunsaturated fatty acid content, which is prone to peroxidation. This process disrupts antioxidant signaling and produces secondary byproducts that can oxidize sperm proteins and lipids [1]. The ability of antioxidants to reduce OS and preserve membrane integrity during the cryopreservation process has been studied across various animal species [5].

Pomegranate (*Punica granatum*) has been traditionally valued in the Middle East for treating various health conditions [6]. Its juice is rich in vitamins E, C, and A, as well as polyphenolic compounds, which are known for strong antioxidant effects. Studies have investigated the antioxidant effects of pomegranate seeds and juice on sperm quality in several species [7,8]. However, the medicinal use of pomegranate seed oil is restricted by challenges such as low solubility and bioavailability, leading to rapid metabolism and limited absorption. To overcome these limitations, researchers have developed various formulations of pomegranate oil nano-emulsions (PO-NE).

The field of nanotechnology has seen remarkable progression and extensive growth offering several potential applications. Lipophilic nanoparticles, such as astaxanthin and the bioactive components in pomegranate, effectively interact with biological systems due to their large surface area and nanoscale size. Studies have shown that delivering certain drugs through nanoparticles can significantly enhance their absorption and bioavailability [9]. Previous studies have shown that nanoparticles can improve semen quality and post-thaw viability by protecting sperm from cryo-damage and OS [9]. While pomegranate extracts and juice have been shown to improve testis function and sperm motility under OS in cryopreserved semen for several species [7,8,10], no studies have specifically examined PO-NE in cryopreserved bovine semen. Understanding sperm ultrastructure and cryo-injury mechanisms could enhance cryopreservation protocols. With PO-NE’s stability, high bioavailability, and nanoscale size, we propose it may offer protective benefits for bovine sperm during cryopreservation. This study aims to assess the impact of adding PO-NE to the freezing medium of cattle semen on post-thaw sperm quality, sperm apoptosis, ultrastructural changes, and the redox balance in seminal plasma.

## MATERIALS AND METHODS

### Preparation of pomegranate oil nanoemulsion

Pomegranate oil was obtained from Pure Life Company, located in Giza, Egypt. A single-layer emulsion of pomegranate oil in water was prepared as described by El-Raghi AA et al [11]. Initially, 2 mL of pomegranate oil was mixed with the surfactant (Tween 80) at 25 °C using a magnetic stirrer. Water (10 mL) was then gradually added at a steady rate of 1.0 mL/min. The resulting emulsion was sonicated for 30 minutes and subsequently homogenized for 5 minutes with an ultrasonic probe (Serial No. 2013020605, Model CV334; Sonics & Materials, Inc., Newtown, CT, USA) attached to a homogenizer (Sonics Vibra-cell VC 505; Sonics & Materials, Inc., Newtown, CT, USA) at amplitude: 60%, and pulser: 1 s ON/1 s OFF.

### Properties of pomegranate oil nanoemulsion

The ultrastructure of the freshly prepared PO-NE was examined using a transmission electron microscope ([TEM] JEOL JEM-2100; JEOL Ltd., Tokyo, Japan) operating at 80 kV. Image acquisition and analysis were conducted using Digital Micrograph and Soft Imaging Viewer software (Gatan Microscopy Suite, version 2.11.1404.0) [12].

### Animal management and semen collection

In this study, seven healthy, fertile Holstein Friesian bulls, aged 4 to 6 years, were selected from a private farm in Dakahlia Governorate, Egypt, and maintained under standard care with veterinary supervision. Semen samples were collected weekly for seven weeks using prewarmed artificial vaginas, yielding a total of 49 ejaculates assessed over seven runs. Each ejaculate was immediately qualified under phase contrast microscope (100×). Only ejaculates that met specific criteria: sperm cell concentration ≥8×10^8^ /mL, abnormalities ≤15%, motility ≥75%, and viability ≥80% were pooled to eliminate the bull effect and to ensure sufficient semen for each replicate. In total, 30 ejaculates met these criteria. These pooled samples were then used for subsequent experimental procedures.

### Extender preparation and experimental design

The bovine semen extender buffer was prepared according to the method outlined by Khalil et al [5]. The sperm were diluted with the prepared extender at a 1:10 ratio (sperm to extender), starting with an initial sperm concentration of 80×10^6^ spermatozoa/mL. The extender was aliquoted into four 15 mL test tubes. The first test tube, without any additives, was labeled as the control (PO-NE0). The other test tubes were labeled as PO-NE1, PO-NE2, and PO-NE4, each supplemented with different concentrations of PO-NE (1, 2, and 4 μg/mL, respectively). Each test tube was further divided into 40 straws for subsequent analyses.

### Freezing and thawing

After dilution, the mixture was gently stirred and maintained at 37°C in a water bath. The diluted semen samples were subsequently incubated at 5°C for 4 hours to allow equilibration. Afterwards, the semen was loaded into 0.25 mL French straws (IVM Technologies, L’Aigle, France). The straws were exposed to smoke by holding them 4 cm above the liquid nitrogen surface for 10 minutes, then fully immersed for cryopreservation. After one month of cryopreservation, thawing was performed by placing the straws in a water bath at 37°C for 30 seconds to prepare for subsequent assessments and analyses, as described by Abdelnour et al [13].

### Evaluation of frozen-thawed semen characteristics

After thawing, the sperm progressive motility (PM) was assessed using a phase-contrast microscope (DM 500; Leica, St. Gallen, Switzerland) equipped with a warm stage (37°C) at 10x magnification. A 10 μL sample of the diluted bovine semen was placed on a prewarmed slide, covered with a coverslip, and analyzed by professional investigator in a blind manner. Each semen sample was evaluated three times, following the procedure described by Khalil et al [5]. The Eosin/Nigrosine staining method was used to assess sperm viability. For analysis, 10 μL of each semen sample were incubated at 25°C for 2 minutes with 10 μL of the eosin/nigrosine solution. A minimum of 200 sperm cells were examined under a light microscope at 400x magnification. Sperm cells that were pink-stained were considered non-viable, while unstained cells were regarded as viable. Additionally, sperm morphology was assessed for abnormalities in tail and head morphology using the method outlined by Samplaski et al [14]. The abnormalities assessed included loose heads, double heads, microcephalic heads, pear-shaped heads, round short heads, coiled tails, broken tails, and double tails. To assess sperm membrane integrity, the hypo-osmotic swelling test, was conducted. This test involved examining 200 sperm cells for swelling in a hypo-osmotic solution. Sperm cells with coiled or swollen tails were regarded as having intact plasma membranes.

### Computerassisted sperm analysis

Live images of detailed sperm motility parameters were analyzed using Computer-Assisted Sperm Analysis (CASA) software. The images were captured using an Olympus BX microscope (Hamburg, Germany) connected to a high-speed digital camera, which recorded at 60 frames per second and 60 Hz under ×4 dark-field illumination. This system was integrated with the CASA software, which analyzed approximately 1,500 spermatozoa across all experimental groups. The software recorded several motion parameters to quantify key metrics, including PM (%), distance average path (DAP, μm), distance curved line (DCL, μm), distance straight line (DSL, μm), velocity average path (VAP, μm/s), velocity curved line (VCL, μm/s), velocity straight line (VSL, μm/s), straightness (VSL/VAP, %), linearity (VSL/VCL, %), wobble (VAP/VCL, %), amplitude of lateral head displacement (ALH, μm), and beat cross frequency (BCF, Hz).

### Apoptosis assessment via flow cytometry

Apoptosis in frozen-thawed bovine spermatozoa was evaluated using flow cytometry and Annexin V staining. Semen samples from all groups were centrifuged and resuspended in 2 mL of binding buffer, followed by thorough mixing. A 100 μL aliquot of the suspension was combined with 5 μL of propidium iodide (PI) and 5 μL of Annexin V (fluorescein isothiocyanate-labeled). The sperm samples were then incubated in the dark for 15 minutes. After incubation, the samples were diluted with 200 μL of binding buffer for further analysis. Apoptosis was assessed using a flow cytometer (Accuri C6 Cytometer, BD Biosciences, San Jose, CA, USA) and Becton Dickinson software, according to the protocol outlined by Masters et al [15]. The percentages of PI-negative/positive (PI−/PI+), Annexin V-negative/positive (A−/A+), and double-positive cells were determined. Based on the staining patterns, sperm were classified into four categories as described by Peña et al [16]: 1) Viable cells: These cells show no fluorescence signal and have intact membrane function (A−/PI−). 2) Early apoptotic sperm cells: These cells are viable but show Annexin V labeling without PI staining (A+/PI−). 3) Late apoptotic sperm cells: These non-viable cells exhibit both Annexin V labeling and PI staining, indicating compromised membranes (A+/PI+). 4) Necrotic sperm cells: These non-viable cells are labeled with PI but lack Annexin V, suggesting damaged and permeable membranes (A−/PI+).

### Ultrastructure analysis of cryopreserved spermatozoa

Frozen-thawed semen was fixed for 2 to 4 hours in a 2% glutaraldehyde solution in phosphate-buffered saline (PBS). Following fixation, the samples were washed three times with PBS by centrifugation at 4°C for 15 minutes each. After washing, the semen samples were post-fixed with 1% osmium tetroxide solution at 4°C for 2 hours. The samples were then dehydrated through a graded series of ethanol solutions. Following dehydration, the specimens were embedded in Epon resin for polymerization. Ultrathin sections were prepared using an RMC ultramicrotome and stained with uranyl acetate and lead citrate. Finally, the sections were examined under a TEM (JEM-2100; JEOL, Tokyo, Japan).

### Oxidative biomarker assays

Frozen-thawed semen samples were centrifuged at 4,025 ×g for 10 minutes. After centrifugation, the extender was separated from the samples and stored at −20°C. The concentrations of total antioxidant capacity (TAC), malondialdehyde (MDA), and nitric oxide (NO) were examined in the extender as described in previous studies [17–19]. The linearity limits for MDA, TAC, and NO were determined to be 100 nmol/mL, 2 mM/L, and 200 μmol/L, respectively, which define the highest concentrations that can be accurately measured using the spectrophotometer. The assays were performed using commercial kits from Biodiagnostic Company (Giza, Egypt), following the manufacturer’s instructions. Superoxide dismutase (SOD) activity was assessed using ELISA kits from Beyotime Biotechnology (Shanghai, China), according to the manufacturer’s guidelines.

Nuclear factor kappa B (NF-κB) levels were evaluated through ELISA kits obtained from Beyotime Biotechnology in Shanghai, China, following the provided instructions. The concentration of H_2_O_2_ was measured calorimetrically at 510 nm utilizing the Phenol Red method as described by Maia et al [20] with a commercial kit (HP 25, Bio Diagnostic, Egypt).

### Total RNA extraction and mRNA quantification

Total RNA was extracted from post-thawed cryopreserved spermatozoa using TRIzol reagent (Invitrogen Co., Waltham, MA, USA) following the manufacturer’s instructions. RNA concentration and quality were assessed using a Nanodrop spectrophotometer (Nanodrop 2000; Thermo Fisher, Waltham, Massachusetts, USA) by measuring the absorbance ratio at A260/280. cDNA synthesis was performed with the Prime Script RT Reagent Kit (Takara, Dalian, China), according to the provided guidelines. The expression of anti-apoptotic *Bcl-2* (B-cell lymphoma-2), pro-apoptotic *Bax* (BCL2-associated X), and apoptotic *caspase 3* was analyzed by reverse transcription quantitative polymerase chain reaction (RT-qPCR), with β-actin used as the housekeeping gene. The primer sequences are listed in Table 1. RT-qPCR was conducted using ChamQ SYBR qPCR Master Mix (Vazyme Biotech, Nanjing, China), with reaction conditions set to 30 seconds at 95°C, followed by 40 cycles of 95°C for 5 seconds and 60°C for 30 seconds. The comparative gene transcript level was quantified using the 2^−ΔΔCt^ method, with β-actin serving as the reference gene for normalization.

### Statistical analysis

Data editing was performed using Microsoft Excel, version 16. The Shapiro-Wilk test was applied to assess the normality of the data [21]. To determine the effect of treatments, a one-way ANOVA was conducted with a significance level set at α = 0.05. The statistical model used for analysis was: Y**_ij_** = μ+TRT**_i_**+e**_ij_**, where Y**_ij_** represents the observations, μ is the overall mean, TRT indicates the effect of PO-NE (i, 1 to 4), and e**_ij_** represents random error. When a significant effect was detected, Tukey’s HSD test was applied for pairwise mean comparisons, with the significance level set at p<0.05.

## RESULTS

### Characterization of pomegranate oil nanoemulsion formation

**Figure 1:**
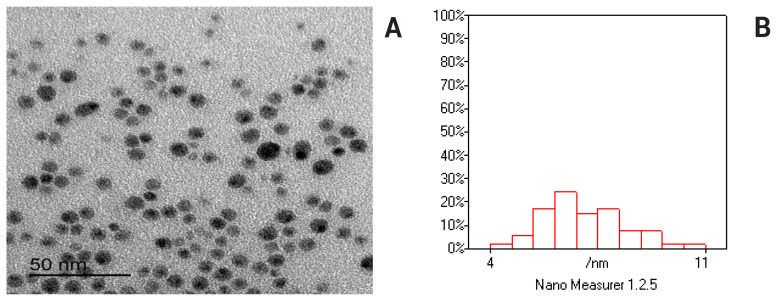
The TEM image of PO-NE (A) reveals morphology with almost spherical particles, while the histogram (B) indicates that most particles are within the 4 to 11 nm range. TEM, transmission electron microscopy; PO-NE, pomegranate oil nano-emulsions.

The TEM image for PO-NE is presented in Figure 1A. The figure displays nearly spherical particles morphology, Nano-emulsion oils with no or little aggregation identified. The mean of particles size was 4 to 11 nm (Figure 1).

### Effects of pomegranate oil nano-emulsions on post-thawed sperm characteristics

Table 2 shows that adding different concentrations of pomegranate oil to the freezing extender significantly enhances bovine sperm PM, viability, and membrane integrity after thawing compared to the control group (p<0.05), with the highest improvements observed in the PO-NE4 group. However, there were no significant differences in the percentage of abnormal sperm, primarily consisting of coiled and bent tails between the control group and all PO-NE treated groups (p>0.05).

### Effects of pomegranate oil nano-emulsions on kinematic parameters of post-thawed bull spermatozoa

In this study, all kinematic parameters except straightness, linearity, and wobble were significantly influenced by PO-NE treatments (p-values ranged from 0.0415 to <0.0001). Parameters such as PM, DCL, VAP, VCL, VSL, DAP, DSL, ALH, and BCF showed notable improvements in all treated groups compared to the control (p<0.05), with the most substantial enhancements in the PO-NE4 group. No significant differences were observed between the PO-NE1 and PO-NE2 treated groups regarding all the motion parameters (p>0.05; Table 3).

### Impact of pomegranate oil nano-emulsions on antioxidant capacity, nitric oxide, and nuclear factor kappa B in post-thawed bull spermatozoa

Table 4 presents the redox status analysis of bovine sperm following freezing. All PO-NE-treated groups showed a significant increase in TAC and SOD levels compared to the control group (p<0.05). In comparison to the control group, the PO-NE2 and PO-NE4 groups exhibited significantly reduced levels of MDA, hydrogen peroxide, NO, and NF-κB (p<0.05).

### Effects of pomegranate oil nano-emulsions on sperm apoptosis and apoptotic genes of post-thawed bovine sperms

**Figure 2:**
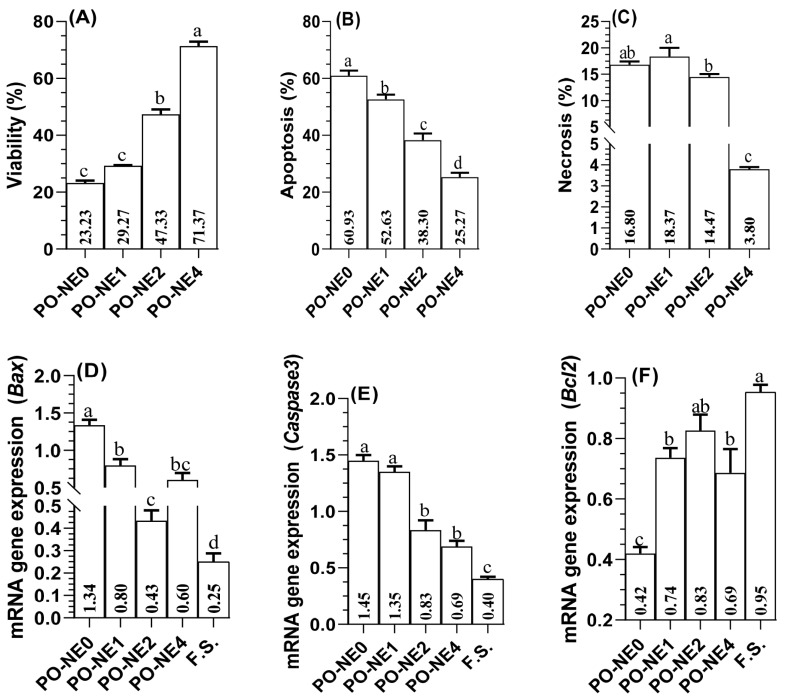
Effect of supplementing freezing extender with different levels of pomegranate oil nano-emulsion on sperm viability (A) apoptosis (B), and necrosis (C), mRNA expression of apoptotic related genes using RT-qPCR in bovine spermatozoa including (D) pro-apoptotic *BAX* (BCL2-associated X), (E) *Caspase* 3, and (F) anti-apoptotic *BCL*-2 (B-cell lymphoma). PO-NE0, PO-NE1, PO-NE2, and PO-NE4 correspond to concentrations of 0, 1, 2, and 4 μg of pomegranate oil nano-emulsion per milliliter, respectively. Results are expressed as mean±standard error. Sperm viability, apoptosis, and necrosis were assessed using a flow cytometry system. A total of 10 straws (n = 10) were used for flow cytometry and n = 5 for gene expression. ^a–d^ Bar with different letters differ (p<0.05). F.S, fresh semen; RT-qPCR, reverse transcription quantitative polymerase chain reaction; TEM, transmission electron microscopy.

In Figures 2A–2C, the correlation between pomegranate levels in the bovine freezing extender and the parameters of viability, apoptosis, and necrotic cells is illustrated. The present results demonstrate a direct relationship between pomegranate concentration in the freezing extender and viability (p< 0.05; Figure 2A), showing a linear increase with higher pomegranate levels. In contrast, the incidence of apoptosis displayed a linear decrease (p<0.05; Figure 2B). The count of necrotic cells was notably decreased in both of PO-NE2 and PO-NE4 treated groups compared to the control group. However, there were no significant differences between the control group and the PO-NE1 treated groups (p>0.05; Figure 2C). Figure 2D–2F exhibit the transcript levels of apoptosis-related proteins confirmed through RT-qPCR. The supplementation of 2 or 4 μg/mL of PO-NE led to a pronounce decrease in the mRNA expression of the pro-apoptotic genes *Bax* (Figure 2D) and *caspase* 3 (Figure 2E) in the bovine sperm (p<0.05), with the greatest reduction observed in fresh semen. Conversely, the inclusion of all concentrations of PO-NE significantly up regulated the expression of the anti-apoptotic gene *Bcl*-2 compared to the control group, with the highest expression in fresh semen. No significant differences were found between fresh semen and the PO-NE2 treatment (p<0.05; Figure 2F).

### Sperm ultrastructure

**Figure 3:**
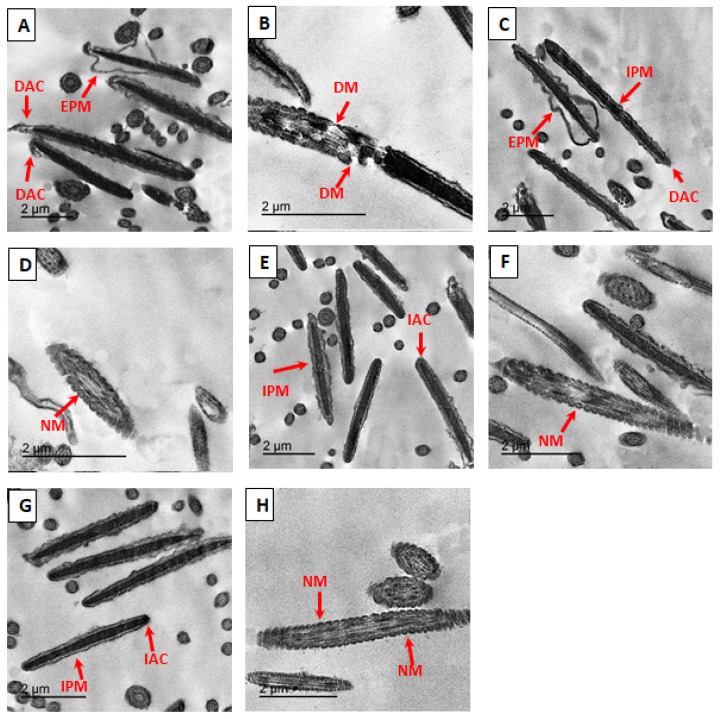
The impacts of supplementing extender with pomegranate oil nano-emulsion (PO-NE) at various concentrations extender on ultra-structural of frozen-thawed bovine spermatozoa. (A,B) 0 μg g/mL (PO-NE0), (C,D) 1 μg g/mL (PO-NE1), (E,F) 2 μg g/mL (PO-NE2), (G,H) 4 μg g/mL (PO-NE4). DAC, damaged acrosomal; EPM, extended plasma membrane; DM, damaged mitochondria; IPM, intact plasma membrane; NM, normal mitochondria; IAC, intact acrosomal; RT-qPCR, reverse transcription quantitative polymerase chain reaction; TEM, transmission electron microscopy.

The effects of supplementing PO-NE on the ultrastructure of post-thawed bovine spermatozoa were evaluated at various concentrations. In the control group (PO-NE0, Figures 3A, 3B), numerous alterations in the cell membrane were observed, ranging from discontinuity to complete damage, including extended plasma membrane, complete plasma membrane damage, damaged acrosome in Figure 3A, necrotic chromatin in Fig. 3B, and mitochondria with distorted cristae. In the PO-NE1 group (Figures 3C, 3D), abnormalities similar to the control were detected, including sperm plasma membrane damage, damaged acrosome, and normal mitochondria, along with condensed chromatin. Meanwhile, the PO-NE2 (Figures 3E, 3F) and PO-NE4 (Figures 3G, 3H) groups exhibited limited changes in sperm ultrastructure.

## DISCUSSION

During cryopreservation, spermatozoa undergo various stresses that can impair their fertilizing ability. As a result, numerous studies have aimed to improve sperm quality and reduce the harmful effects of OS. To achieve this goal researchers have studied the effect of using natural antioxidants [22] and nanoparticles [9] in freezing media to address this issue. However, no studies have yet investigated the use of PO-NE in cryopreserved bovine semen. It is widely recognized that particles with low particles size lack the repulsive forces necessary to prevent particle flocculation or aggregation. The particles size of PO-NE was between 4 and 11 nm, indicating strong stability. The delicate balance between OS and antioxidants enzymes is crucial for sperm functions, as an imbalance can lead to notable loss of functionality. To mitigate the harmful effects of OS that can damage sperm cells during cryopreservation, the addition of PO-NE at different concentrations to bovine semen extender was evaluated.

Post-thawed sperm quality stands out as a dynamic factor closely correlated with sperm motility, viability, integrity, and overall fertility. CASA represents a comprehensive and precise technique for assessing various aspects of sperm kinematic parameters. The results of this study reveal a significant improvement in post-thaw sperm characteristics, including PM, viability, and membrane integrity, when 2 or 4 mM of PO-NE was added to a tris-based extender. All kinematic parameters, such as VSL, VCL, VAP, ALH, and BCF, also showed enhanced levels at these concentrations. Limited scientific reports at the molecular level exist on the protective effects of pomegranate oil on frozen spermatozoa for direct comparison with our findings. However, our results align with previous research on various species that utilized raw pomegranate seeds and juice [7,8,10], demonstrating positive effects on post-thaw sperm motility and viability. The observed improvements may be attributed to the antioxidants present in pomegranate seed oil, including polyphenols, anthocyanins, ellagic acid, gallic acid, vitamins C and E, and punicalagin [23]. Unlike prior studies that used pomegranate in its raw seed form or as juice, this study employed it as a nanoemulsion, enhancing its solubility, stability, and bioavailability. This enhancement promotes better metabolism and absorption, allowing for lower yet more effective doses.

It has been demonstrated that the antioxidant properties of PO-NE may be associated with the scavenging of reactive oxygen species (ROS) during cryopreservation, as well as an improved antioxidant enzyme profile in sperm cells. The antioxidant defense system is vital for protecting spermatozoa from OS and peroxidative damage, both of which are major contributors to spermatogenic dysfunctions [24]. In the absence of this defense mechanism, ROS can lead to elevated levels of abnormal sperm cells, impaired sperm motility, and decreased spermatogenic cell density [25]. The freezing process can exacerbate the generation of ROS [26], leading to changes in sperm membrane structure and function, as well as disruptions in antioxidant defense systems [27], including reduced activity of antioxidant enzymes like glutathione peroxidase and SOD [13]. To counteract the harmful effects of ROS, seminal plasma contains an antioxidant system that is crucial for protecting sperm cells [8]. Unfortunately, spermatozoa have limited antioxidant capacity compared to somatic cells, making them less capable of defending against ROS [3]. Supplementing the freezing and thawing medium with natural antioxidants may help enhance the viability and fertilizing potential of frozen-thawed spermatozoa from farm animals by reducing OS and improving antioxidant enzyme activity [13]. In the present study, adding PO-NE at doses of 2 or 4 μg/mL to the freezing extender led to a significant reduction in the levels of MDA and H_2_O_2_, while significantly increasing TAC and SOD activity. Several studies have examined the effectiveness of adding various natural antioxidants to extenders to protect spermatozoa from the harmful effects of ROS [28]. Türk et al [7] reported that pomegranate fruit contains exceptionally high antioxidant concentrations, measuring 11.33 mmol/100 g. It is also known that the phenolic compounds and flavonoids in pomegranate have antioxidant effects that are significantly stronger than those of vitamins C and E [8]. The antioxidant capacity of pomegranate is attributed not only to its vitamin C and E content but also to other antioxidant-rich compounds such as flavonoids and tannins [29]. Moreover, fresh pomegranate contains approximately 1.5% pectin, 10% total sugars, ascorbic acid, flavonoids, polyphenols, and key amino acids such as aspartic and glutamic acid [30]. Gangwar et al [31] showed that vitamin C, at a concentration of 56.78 mmol/L, can act as a potent antioxidant in semen extenders during the standard freezing process, enhancing post-thaw recovery of buck semen. Naturally found in seminal plasma, vitamin C helps neutralize and reduce the harmful effects of free radicals, including lipid peroxidation [31]. Adding vitamin C to the extender may improve sperm function by mitigating cell damage through its continuing radical-scavenging activity.

The high content of polyunsaturated fatty acids (PUFAs) in sperm membranes makes them particularly susceptible to lipid peroxidation, especially in cattle semen. Lipid peroxidation damages the lipid components of sperm membranes, leading to axonemal damage, impaired intracellular energy production, increased mid-piece morphological defects, and ultimately reduced sperm viability [32]. Both the acrosome and plasma membranes are crucial in regulating extracellular exchanges [33]. The lipids that make up the sperm membrane structure are vital for motility, viability, and cryo survival. In the present study, significant improvements in the integrity of both plasma and acrosome membranes were observed in cryopreserved semen samples treated with 4 mg of PO-NE. The enhancement in post-thaw membrane integrity can be attributed to the antioxidant properties of compounds in pomegranate oil, which reduced lipid peroxidation, as evidenced by a significant decrease in MDA levels, and improved plasma membrane integrity. These findings are consistent with similar studies on cattle and goat semen [8].

NO can negatively affect sperm motility and viability. This harmful effect is linked to NO’s role as an inflammatory mediator, which is often triggered by subclinical or chronic infections. Additionally, NO has been shown to reduce cellular adenosine triphosphate (ATP) levels by inhibiting the enzymatic functions of the electron transport chain, which is crucial for ATP production [34]. Since sperm motility depends on mitochondrial ATP production, any reduction in ATP synthesis can result in inadequate energy levels, impairing sperm movement. Increased NO levels have been shown to correlate with poor sperm function in conditions like teratospermia, leukocytoasthenospermia, leukocytospermia, normospermia, and asthenospermia in infertile individuals. NF-κB, a key transcription factor, links inflammation, OS, and apoptosis. Upon activation by OS, NF-κB upregulates inducible nitric oxide synthase (iNOS), leading to higher NO production. Elevated NO levels, in turn, stimulate NF-κB, initiating an inflammatory signaling cascade that triggers the release of various cytokines [35]. In this study, adding 2–4 μg of PO-NE to the freezing medium significantly reduced the levels of NF-κB and NO, resulting in improved sperm motility and velocity after thawing. These findings suggest that NO may play a role in the functionality of cryopreserved bull spermatozoa.

Apoptosis is considered a major cause of DNA damage in sperm during spermatogenesis. Previous studies have indicated a correlation between elevated levels of apoptotic sperm and reduced fertility in farm animals [36]. While the potential effects of PO-NE on cryopreserved semen remain unclear, the current study highlights the protective role of PO-NE in minimizing the incidence of apoptotic spermatozoa. These findings are consistent with previous studies showing that adding natural antioxidants to mouse and rabbit semen can significantly lower the number of apoptotic spermatogonia stem cells [36]. Additionally, Soliman et al. [37] reported that pomegranate juice extract could reduce apoptosis, alleviate OS, and promote autophagy in mouse spermatogonia cells exposed to acrylamide. Similarly, Ali et al [38] demonstrated that pomegranate peel extract mitigates furan-induced testicular damage by influencing apoptosis, steroidogenic enzymes, and OS. However, the exact mechanisms through which PO-NE induces autophagy to protect sperm during cryopreservation require further investigation, as this was not explored in our study.

Apoptosis is regulated by various genes that control cell death, with the Bcl-2 family being particularly important. The Bcl-2 gene, initially identified through a chromosomal translocation in B-cell follicular lymphoma, can block multiple apoptotic signals [39]. Key members of this family, including Bcl-2 and Bax, play crucial roles in apoptosis regulation [40]. Additionally, caspases, especially caspase-3, are central to the apoptosis process, with caspase-3 acting as a critical protease that cleaves essential cellular proteins [37]. In this study, supplementing bovine semen with 2 or 4 μg/mL of PO-NE resulted in the downregulation of caspase-3 and Bax genes, minimizing their expression in fresh semen. Additionally, a significant upregulation of the Bcl-2 gene was observed. No significant difference was detected between semen supplemented with 2 μg/mL of PO-NE and fresh semen. These results align with previous research showing increased expression of Bcl-2 and decreased expression of Bax and caspase-3 in response to pomegranate peel or juice extracts [37,38]. Our findings suggest that pomegranate extract reduces programmed cell death by mitigating OS and preserving membrane integrity. Mitochondrial damage triggers cytochrome c release, activating the apoptotic cascade, which involves genes such as caspase-3 and Bax. This finding is further supported by ultrastructural investigations, which confirm the reduction of harmful effects at the cellular level. The decrease in caspase-3 levels and upregulation of the Bcl-2 gene in the PO-NE supplemented group suggest that the apoptotic cascade is not fully activated, indicating that the sperm cells are being protected from apoptosis. These results confirm the protective effect of PO-NE, as it mitigates OS by reducing NO and MDA levels, as previously indicated.

To gain deeper insights into the cryoprotective effects of PO-NE on bovine post-thawed semen, we conducted a study using TEM to examine sperm ultrastructural changes. Our results show that incorporating PO-NE into the freezing media helps maintain sperm health and functionality by minimizing the harmful effects such as acrosomal nuclear membrane breakage, damage of acrosome and plasma membrane, vesicular-swollen mitochondria, and disrupted chromatin across all treated groups. Notably, in the PO-NE4 treatment group, after thawing, the plasma membrane successfully enveloped the middle piece (normal mitochondria), and nucleus. This is consistent with the findings of [5], which demonstrated that sperm functioning was improved, and the ultrastructure was maintained in buffalo bulls by adding nanoparticles to freezing extenders.

## CONCLUSION

The observed improvement in sperm viability, motility, and velocity is likely due to the antiapoptotic effects of PO-NE. Adding 4 mM of PO-NE to the freezing extender enhances sperm motility, viability, and kinematic parameters, while preserving both acrosomal and plasma membrane integrity in frozen-thawed bovine semen. Additionally, PO-NE supplementation reduces apoptotic gene expression, strengthens antioxidative defenses, and minimizes ultrastructural damage during cryopreservation. Although the exact mechanisms of PO-NE in cryopreserved semen remain unclear, the results suggest its protective role in maintaining sperm ultrastructure and reducing apoptosis, thus improving sperm quality.

## Figures and Tables

**Table 1:** Primers of genes (Bcl-2, Caspase-3, Bax, and β-actin) for real time PCR

Gene	Sequence (5′–3′)	Amplicon size (bp)
*Bcl-2*	F: 5′ ATAGGC ACCCAG GGTGAT 3′ R: 5′ GTG TGTGGAGAG CGTCAAC 3	114
*Caspase-3*	F: 5′-CTG TGG CATTG AGACAG -3′ R: 5′-CCCGTCCTTTGA ATT TC-3′	132
*Bax*	F: 5′-TTG CTTCAG GGTTTC ATCC-3′ R:5′-AGACACT CGC TCAGCTTCT-3′	165
*β-actin*	F: 5′-GGC ACCCA GCACAA-3′ R: 5′-GATCCAC ACG GAG TACT-3	198

PCR, polymerase chain reaction; bp, base pair.

**Table 2:** Effect of adding different concentrations of pomegranate oil nanoemulsion to freezing extender on post-thawed bovine sperm characteristics (%)^1)^

Items	PO-NE 0^2)^	PO-NE1	PO-NE2	PO-NE4	p-values^3)^
PM	35.55±1.30^c^	43.89±0.73^b^	47.22±1.68^b^	56.11±1.82^a^	<0.0001
LIV	35.67±1.33^b^	42.78±0.93^a^	43.44±0.82^a^	44.00±1.42^a^	<0.0001
HOST	33.11±1.45^b^	40.56±1.42^a^	40.44±0.93^a^	42.22±1.43^a^	0.0001
ABN	16.88±0.38	15.11±0.45	15.56±0.60	16.11±0.61	0.1140

1) Results are presented as mean±standard error. A total of 10 straws (n = 10) were used.

2) PO-NE0, PO-NE1, PO-NE2, and PO-NE4 = 0, 1, 2, and 4 μg pomegranate oil nano-emulsion/Ml, respectively.

3) A p-values were calculated by the one way analysis of variance. All sperm characteristics were evaluated using a light microscope.

a–c Values within the same row that have different superscript letters are significantly different (p<0.05; Tukey HSD test).

PM, progressive motility; LIV, livability; HOST, membrane integrity; ABN, abnormality.

**Table 3:** Impact of adding various concentrations of pomegranate oil nanoemulsion to freezing extender on kinematic parameters of post-thaw bovine spermatozoa^1)^

Items	PO-NE0^2)^	PO-NE1	PO-NE2	PO-NE4	p-values^3)^
PM (%)	30.43±0.55^c^	42.38±1.28^b^	41.92±2.66^b^	47.68±0.23^a^	0.0003
DAP (μm)	17.78±0.17^c^	21.78±0.61^b^	22.09±0.72^b^	28.53±0.98^a^	<0.0001
DCL (μm)	23.69±1.01^c^	30.87±1.70^b^	32.13±0.59^b^	39.26±1.12^a^	0.0001
DSL (μm)	13.42±0.11^b^	14.83±0.47^b^	15.08±0.33^b^	18.54±0.73^a^	0.0227
VAP (μm/s)	34.79±0.71^c^	43.50±1.58^b^	45.05±1.22^b^	50.90±2.20^a^	0.0005
VCL (μm/s)	61.77±3.36^b^	77.50±4.28^a^	82.20±1.11^a^	85.29±2.30^a^	0.0018
VSL (μm/s)	23.19±0.54^c^	29.72±1.28^b^	30.93±0.39^b^	34.57±0.97^a^	0.0002
STR (VSL/VAP; %)	66.72±2.27	68.29±0.48	68.71±0.97	68.09±2.62	0.7741
LIN (VSL/VCL; %)	37.81±2.53	38.41±0.51	37.64±0.48	40.56±1.12	0.4872
WOB (VAP/VCL; %)	56.53±1.95	56.26±1.15	54.81±1.26	59.63±0.99	0.1147
ALH (μm)	4.18±0.04^a^	4.45±0.08^a^	4.77±0.04^a^	3.14±0.34^b^	0.0041
BCF (Hz)	15.71±0.26^c^	19.81±0.13^b^	19.78±0.23^b^	25.49±1.97^a^	0.0415

1) Results are presented as mean±standard error. A total of 10 straws (n = 10) were used.

2) PO-NE0, PO-NE1, PO-NE2, and PO-NE4 correspond to 0, 1, 2, and 4 μg of pomegranate oil nanoemulsion per mL, respectively.

3) p-values were calculated by the one way analysis of variance. All motion parameters were evaluated using a Computer-Assisted Sperm Analysis (CASA) device.

a–c Values within the same row marked with different superscripts are significantly different (p<0.05; Tukey HSD test).

PM, progressive motility; DAP, distance average path; DCL, distance curved line; DSL, distance straight line; VAP, velocity average path; VCL, velocity curved line; VSL, velocity straight line; STR, straightness; LIN, linearity; WOB, wobble; ALH, amplitude of lateral head displacement; BCF, beat cross frequency.

**Table 4:** Effect of supplementing freezing extender with different concentrations from pomegranate oil nanoemulsion on redox status, nitrosative biomarker, and nuclear factor kappa B in post-thawed bovine semen^1)^

Items	PO-NE0^2)^	PO-NE1	PO-NE2	PO-NE4	p-values^3)^
TAC (mmol/L)	0.82±0.04^c^	0.94±0.02^b^	1.05±0.39^b^	1.21±0.31^a^	0.0003
SOD (ng/mL)	16.43±0.92^b^	18.33±1.89^b^	28.50±1.53^a^	31.93±1.06^a^	0.0001
MDA (ng/mL)	19.77±2.84^a^	11.23±0.22^b^	9.37±0.74^b^	7.77±1.01^b^	0.0027
H_2_O_2_(mM/L)	0.82±0.01^a^	0.66±0.01^b^	0.43±0.00^c^	0.41±0.01^c^	<0.0001
NO(IU/mL)	39.42±1.41^a^	35.77±1.03^a^	37.03±0.44^a^	30.71±1.89^b^	0.0090
NF-Kb(ng/mL)	16.26±1.03^a^	13.03±0.56^b^	9.90±0.20^c^	8.91±0.36^c^	0.0001

1) Results are presented as mean±standard error. A total of 10 straws (n = 10) were used.

2) PO-NE0, PO-NE1, PO-NE2, and PO-NE4 correspond to concentrations of 0, 1, 2, and 4 μg of pomegranate oil nano-emulsion per milliliter, respectively.

3) A p-values were calculated by the one way analysis of variance.

a–c Values in the same row with different superscripts are significantly different (p<0.05; Tukey HSD test).

TAC, total antioxidant capacity; SOD, superoxide dismutase; MDA, malondialdehyde; H_2_O_2_, hydrogen peroxide; NO, nitric oxide; NF-kB, nuclear factor kappa B.
